# Clinical, radiographic and histomorphometry evaluation of the osteogenic potential of Schneiderian membrane after graftless maxillary sinus augmentation: a randomized controlled clinical trial

**DOI:** 10.1186/s12903-026-07772-2

**Published:** 2026-02-19

**Authors:** Mohamed ElSholkamy, Randa H. Mokhtar, Tarek M. Eltayeb, Inas Helwa, Rehab A. Soliman

**Affiliations:** 1 Oral & Maxillofacial Surgery Department, Faculty of Dentistry, Suez Canal University, Ismailia, Egypt; 2 Oral Histopathology Department, Faculty of Oral and Dental Medicine, Misr International University, Cairo, Egypt; 3 Oral Medicine, Periodontology and Oral Diagnosis Department, Faculty of Oral and Dental Medicine, Misr International University, Cairo, Egypt; 4 Oral & Maxillofacial Surgery Department, Faculty of Oral and Dental Medicine, Misr International University, Cairo, Egypt

**Keywords:** Sinus lifting, Bone augmentation, Graftless sinus elevation, Bioabsorbable screws, Dental implants

## Abstract

**Background:**

To evaluate the osteogenic potential of the Schneiderian membrane following sinus floor elevation using bioabsorbable screws as space maintainers, without the use of graft material.

**Methods:**

Fourteen patients with severely atrophic posterior maxilla were enrolled in the current study. All patients underwent maxillary sinus augmentation via a lateral window approach. Patients were randomly allocated into two groups. In the control group, the sinus cavity was filled with xenograft material. In the intervention group, the Schneiderian membrane was elevated and stabilized using bioabsorbable screws, with no graft material used to fill the space.

**Results:**

Cone beam computed tomography (CBCT) of all patients revealed radiographic bone height improvement in both groups, with the control group showing a greater mean increase. Histomorphometric analysis revealed a tendency toward higher new bone formation in the intervention group, though the difference did not reach statistical significance.

**Conclusion:**

Bioabsorbable screws may serve as effective space maintainers for graftless sinus elevation, allowing for spontaneous bone regeneration beneath the Schneiderian membrane. While conventional grafting with xenograft remains predictable, the bioresorbable screws-supported graftless approach offers a promising alternative in sinus floor elevation.

**Trial registration:**

This study protocol was retrospectively registered on the trial registry “Clinical trials.gov PRS”. ClinicalTrials.gov ID is NCT06766292. Registered on January 5th, 2025.

**Supplementary Information:**

The online version contains supplementary material available at 10.1186/s12903-026-07772-2.

## Introduction

Maxillary sinus augmentation may be performed using a variety of biomaterials, including autografts, allografts, xenografts and alloplastic substitutes, each of which offers a distinct balance of osteogenic, osteoinductive, and osteoconductive properties as well as practical considerations such as donor-site morbidity, availability, and long-term volumetric stability. Autogenous bone grafting has long been regarded as the benchmark for maxillary sinus floor augmentation. However, harvesting autogenous grafts is frequently linked to postoperative complications, including pain, swelling, bruising, and functional limitations at the donor site [1]. To reduce these complications, minimally invasive surgical approaches have been developed, utilizing a variety of grafting materials ranging from allogenic and xenogeneic to alloplastic substitutes [2]. Sinus augmentation is commonly performed by creating a space beneath the Schneiderian membrane and filling it with bone graft materials to facilitate new bone formation. graftless maxillary sinus floor elevation has emerged as a biological alternative to traditional grafting. This method relies on membrane elevation alone, often supported by implant placement or space-maintaining devices such as titanium screws or mesh, eliminating the need for grafting materials [3, 4]. Several experimental and clinical studies have confirmed new bone formation following non-grafted sinus lifting [5–7].

The cornerstone of this approach is the formation of a stable blood clot within the secluded sinus cavity, which acts as a natural autologous scaffold rich in mesenchymal stem cells and signaling factors that trigger osteogenesis. While the innate healing potential of the blood clot is well-documented, recent studies emphasize that the adjunctive use of autologous platelet concentrates, such as Platelet Rich Fibrin and Concentrated Growth Factors, can significantly optimize this regenerative environment. Autologous platelet concentrates provide a higher concentration of growth factors (e.g., VEGF, PDGF) and a denser fibrin matrix that is more resistant to early resorption. By reinforcing the natural clot, APCs not only accelerate new bone formation but also offer superior protection to the sinus membrane and improve early implant stability in the absence of exogenous bone substitutes [8, 9]. 

Nevertheless, guided bone regeneration without grafting has demonstrated effective outcomes by relying solely on stable blood clot formation [10]. One of the main difficulties with this approach lies in preserving the integrity of the newly formed blood clot, which can be compromised by intranasal pressure fluctuations associated with breathing [3].

Recent studies have described the use of a graftless lateral window technique, where the elevated membrane is stabilized using implants placed through residual crestal bone [10, 11]. These investigations demonstrated the Schneiderian membrane’s significant osteogenic potential, even in the absence of grafting materials. Furthermore, various space-maintaining strategies have been employed to prevent membrane collapse into the sinus cavity, including the use of titanium screws, resorbable devices, hollow hydroxyapatite scaffolds, and titanium mesh [12, 13]. These techniques preserve the membrane’s elevated position, promoting stable blood clot formation and supporting bone regeneration during healing [14].

Recent clinical studies and systematic reviews consistently demonstrate that xenograft materials provide long-term volumetric stability and support high implant survival rates in maxillary sinus augmentation. These well-documented outcomes reinforce their role as an appropriate and reliable control material for evaluating new sinus lifting techniques [15–18].

The present study is grounded in a graftless sinus floor elevation technique utilizing space-maintaining devices. Its primary aim is to investigate the predictability of osteogenesis beneath an elevated Schneiderian membrane stabilized with bioabsorbable screws, assessed through radiographic and histologic evaluations.

## Materials and methods

### Sample size calculation

Sample size was calculated using the (PS software) based on Abdelsameaa et al. [19]. As regarding the primary outcome (bone height of control group (xenograft group 6.16 ± 0.49), with estimated intervention group of 15% difference from control (0.924); we found that 6 patients per group will be appropriate sample size with the total number 12 patients (2 groups) the power is 80% and α error probability = 0.05,the effect size = 1.8 N.B: Increased number for anticipated missing data: 12 patients increased to 14 patients to compensate for dropout 15%.

The magnitude of the effect to be detected was estimated as mean and standard deviation of the variable of interest and obtained from the scientific literature.

### Ethical consideration

This study protocol was approved by the research ethical committee at faculty of Dentistry, Suez Canal University with a given IRB number 672/2023. Then, the research protocol was registered on clinicaltrials.gov with a given registration number NCT06766292. A written informed consent was signed by all participants after explaining to them all study’s procedures.

### Study design, grouping and settings

This study is a randomized controlled, double-blinded clinical trial with a parallel-group design that was performed at faculty of Dentistry, Suez Canal University. The study was performed on 14 patients with partially edentulous or free end saddle posterior maxillae which require open sinus lifting procedures and bone augmentation.

### Eligibility criteria

**Fig. 1:**
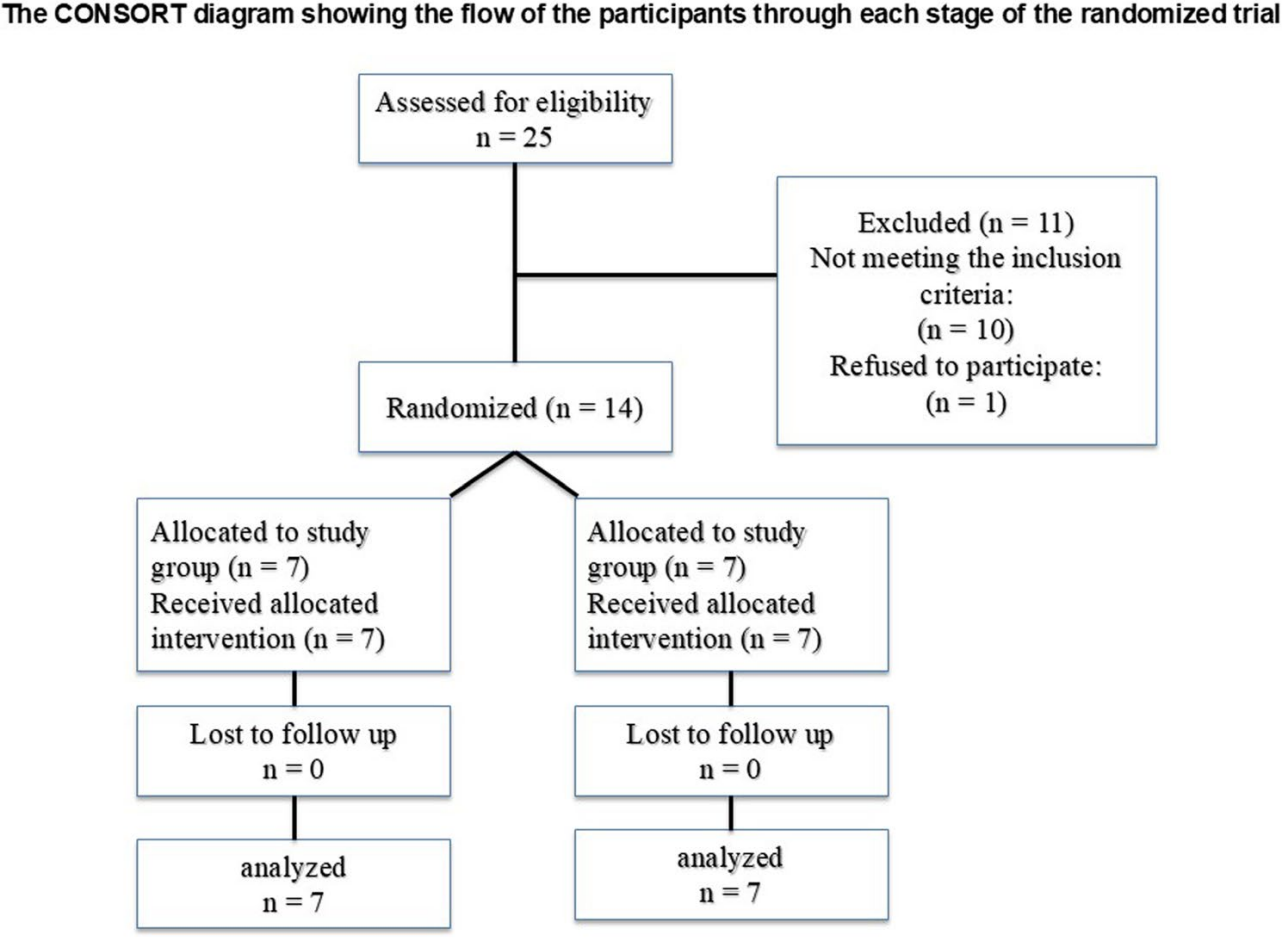
The consort flowchart

Patients presenting with an edentulous posterior maxilla and limited alveolar bone height (≤ 4 mm) were included in the study. All selected individuals had no history of sinus pathology or systemic conditions that could compromise bone healing or negatively impact the predictability of the treatment outcomes. This study was conducted in accordance with the CONSORT guidelines 2010 for the reporting of randomized controlled trials as much as it covers the conducted study (RCTs) (Fig. 1) [20].

### Randomization and blinding

Eligible participants were randomly assigned to either the intervention group, treated with bioabsorbable screws (Inion FreedomScrews™) for tenting the Schneiderian membrane without grafting, or the control group, which received a xenograft alone “Deproteinized bovine bone mineral (DBBM), commercially available as UBGEN (UBGEN S.r.l., Italy)”.

The random allocation sequence was generated by an independent statistician using a computer-generated sequence (www.randomizer.org) with a 1:1 allocation ratio. Participant enrollment was conducted by the principal investigator, who was unaware of the upcoming assignments. Allocation to the intervention groups was performed by a research assistant using sequentially numbered, opaque, sealed envelopes to ensure allocation concealment. Allocation concealment was maintained through sequentially numbered; opaque, sealed envelopes and assignments were disclosed to the principal investigator (RAS) only on the day of surgery. Because of the distinct characteristics of the materials, surgeon blinding was not feasible; however, all surgeries were performed by the same operator under a standardized protocol. Participants, outcome assessors, radiologist, histopathologists, and statistician were blinded to group allocation. Biopsy samples were coded with unique identifiers before histological processing to ensure unbiased evaluation.

### Clinical examination

**Fig. 2:**
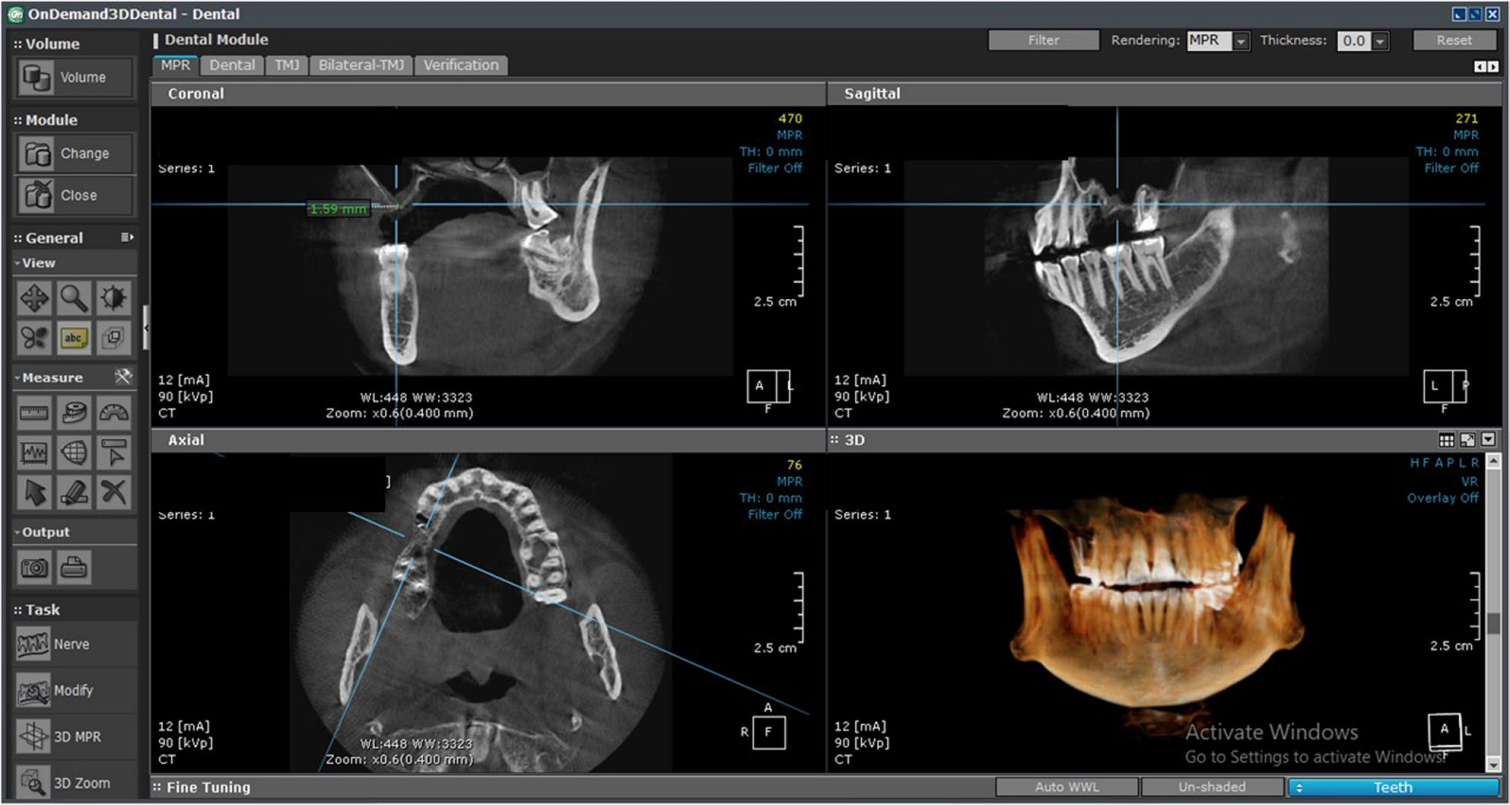
Preoperative CBCT showing the residual bone height in the left maxillary posterior partially edentulous area

A thorough clinical examination was performed to ensure that all enrolled patients met the established inclusion criteria. Preoperative assessment included CBCT with a limited field of view (Papaya 3D Plus, Genoray Co., Korea), using exposure parameters of 90 kV, 12 mA, and an exposure time of 14.5 s. This imaging was used to evaluate and measure the available residual alveolar bone height in the edentulous posterior maxilla (vertical distance between the sinus floor and the alveolar crest) (Fig. 2).

### Surgical procedures

All the surgical procedures were carried out under local anesthesia (Scandonest 2%; Septodont, France) using infraorbital and posterior superior alveolar nerve block with buccal and palatal infiltration. In both groups, the lateral window approach with full thickness mucoperiosteal flap was reflected to expose the lateral wall of the maxillary sinus, followed by sinus floor elevation by open sinus kit (Dentium Advanced Sinus Kit). A bone window was outlined using a round diamond bur no. 8 mounted on straight hand piece with copious irrigation (sterile saline solution) with cautious taken to not penetrate the sinus membrane. The process of bone removal was performed through the cortical plate to expose the Schneiderian membrane without perforation. A round bur (No. 8) was used to outline the margins of the lateral window. The cortical plate within the outlined area was then thinned and gently infractured, allowing it to be reflected inward rather than removed. Once osteotomy was completed, the Schneiderian membrane was carefully elevated to the desired height.

#### Control group (xenograft preparation)

Each 1 gm of xenograft was mixed with 1 mL of sterile saline solution followed by the protocol of graft packed and compacted against inferior walls and to the sides of the antrum until the new available volume created was filled. The lateral window was covered by collagen membrane before flap closure.

#### Intervention group (bioabsorbable screws)

**Fig. 3:**
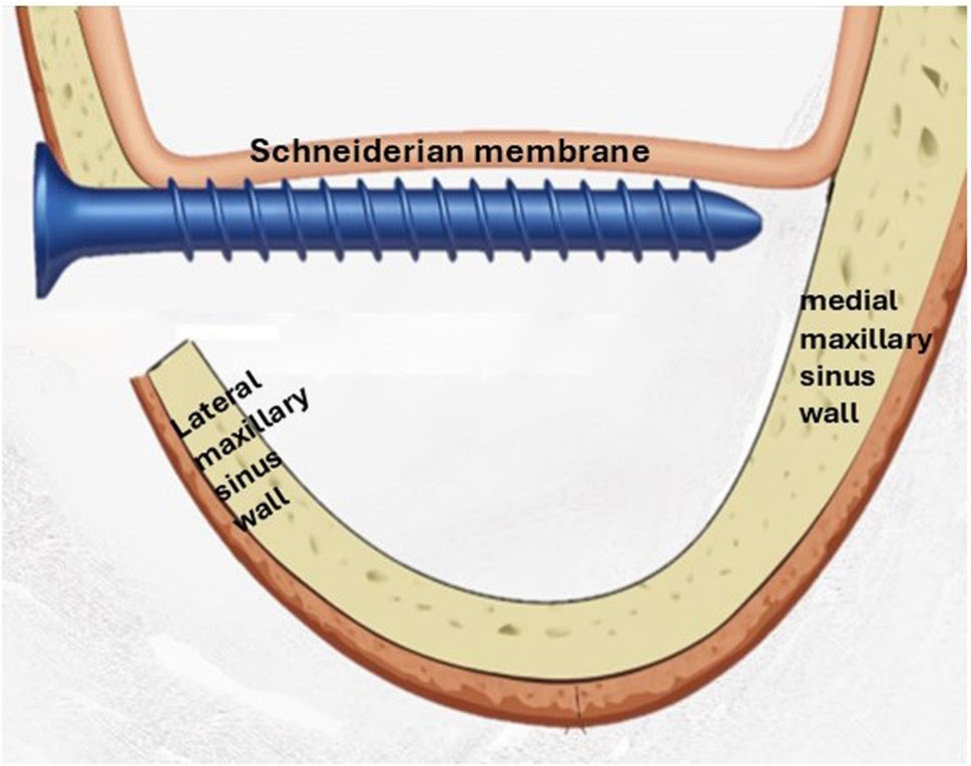
Schematic diagram illustrates the position of the bioabsorbable screws beneath the elevated Schneiderian membrane. (intervention group)

**Fig. 4:**
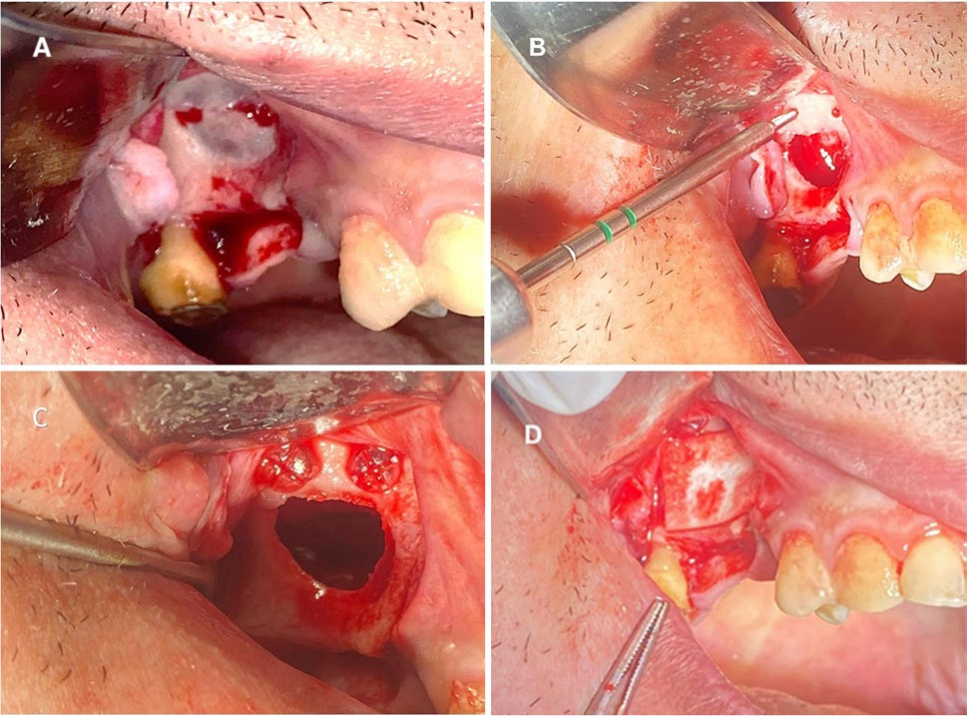
(intervention group)(**A**) Clinical images showing the flap reflection followed by lateral window preparation. **B**,** C** Intraoperative views showing the application of bioabsorbable screws following membrane elevation. **D** Placement of a collagen membrane prior to flap closure

Two screws of 2.0 mm in diameter and 9–11 mm in length were fixed to the lateral wall of the sinus above the superior border of the osteotomy and placed in a lateral-medial direction where the Schneiderian membrane with the bony window was elevated and stabilized (Fig. 3). The distance between the two screws was 5 mm. A limited lateral window osteotomy was prepared intentionally in all cases to avoid a wide lateral window. A collagen membrane was used to close the bone window before flap closure. Then the soft tissue flap was readapted and sutured using continuous and interrupted sutures (3 − 0 resorbable vicryl) (Fig. 4A, B, C &D).

### Postoperative care

Postoperative care included ice packs during the first 6 h and warm saline rinses starting on the second day. Postoperative care included a standardized medication regimen prescribed for all patients: Amoxicillin 875 mg with Clavulanic Acid 125 mg twice daily for one week (*Hibiotic*^®^,* Amoun Pharmaceutical Co.*,* Egypt*), Ibuprofen 600 mg twice daily for one week (*Brufen*^®^,* Abbott Laboratories*,* USA*), and Xylometazoline Hydrochloride 0.1% nasal spray administered 2–3 times every 12 h for one week (*Otrivin*^®^,* Novartis Consumer Health*,* Switzerland*). Patients were advised to avoid activities that increase nasal or sinus pressure, such as nose blowing or sneezing with a closed mouth, for the first 72 h postoperatively. Sutures were removed within 7–10 days, and follow-up evaluations were conducted weekly for one month, then at 3, 6, and 8 months.

### Postoperative radiographic evaluation

**Fig. 5:**
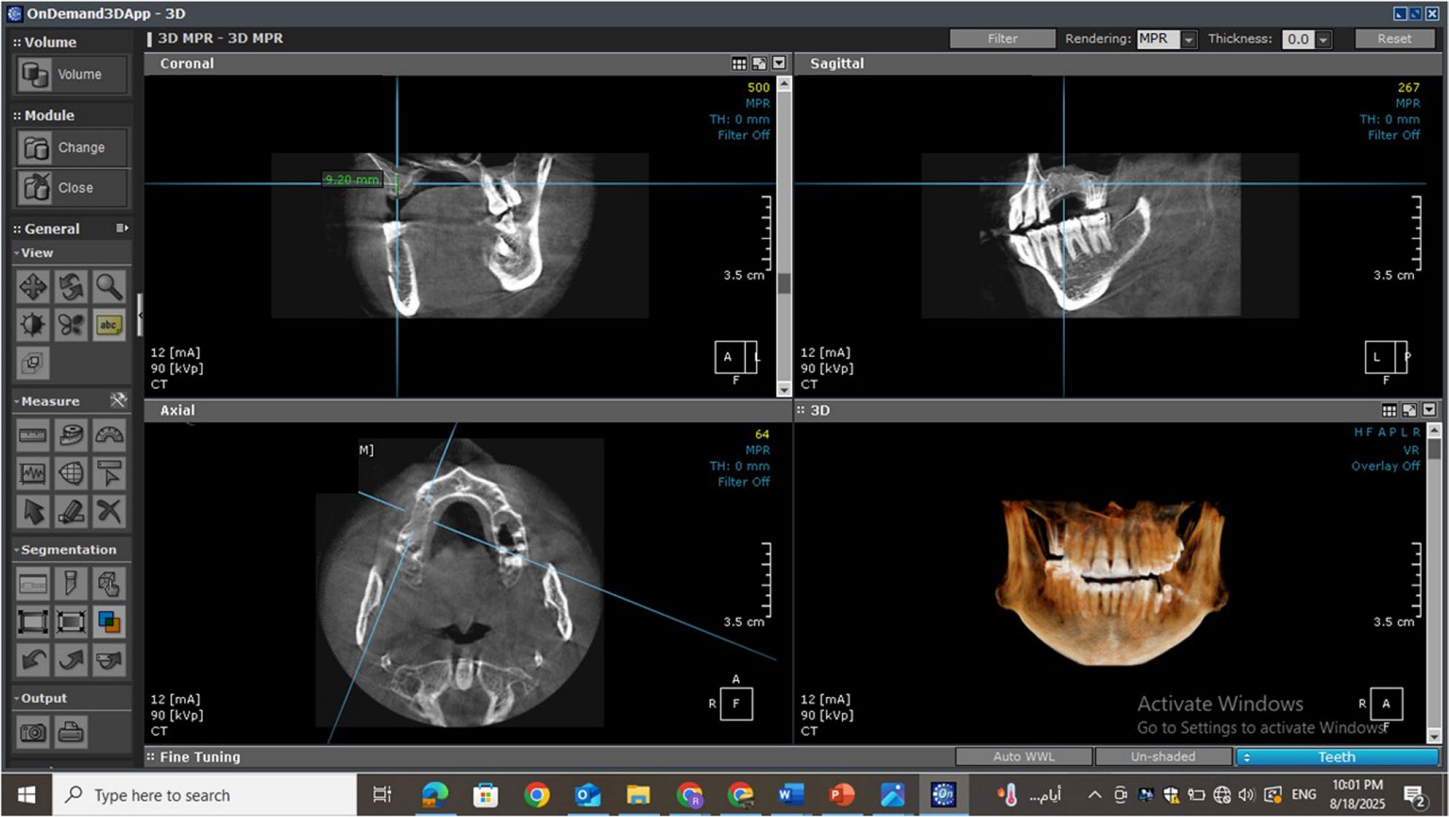
Eight-month postoperative CBCT of the intervention group demonstrating new bone formation beneath the elevated Schneiderian membrane. (intervention group)

**Fig. 6:**
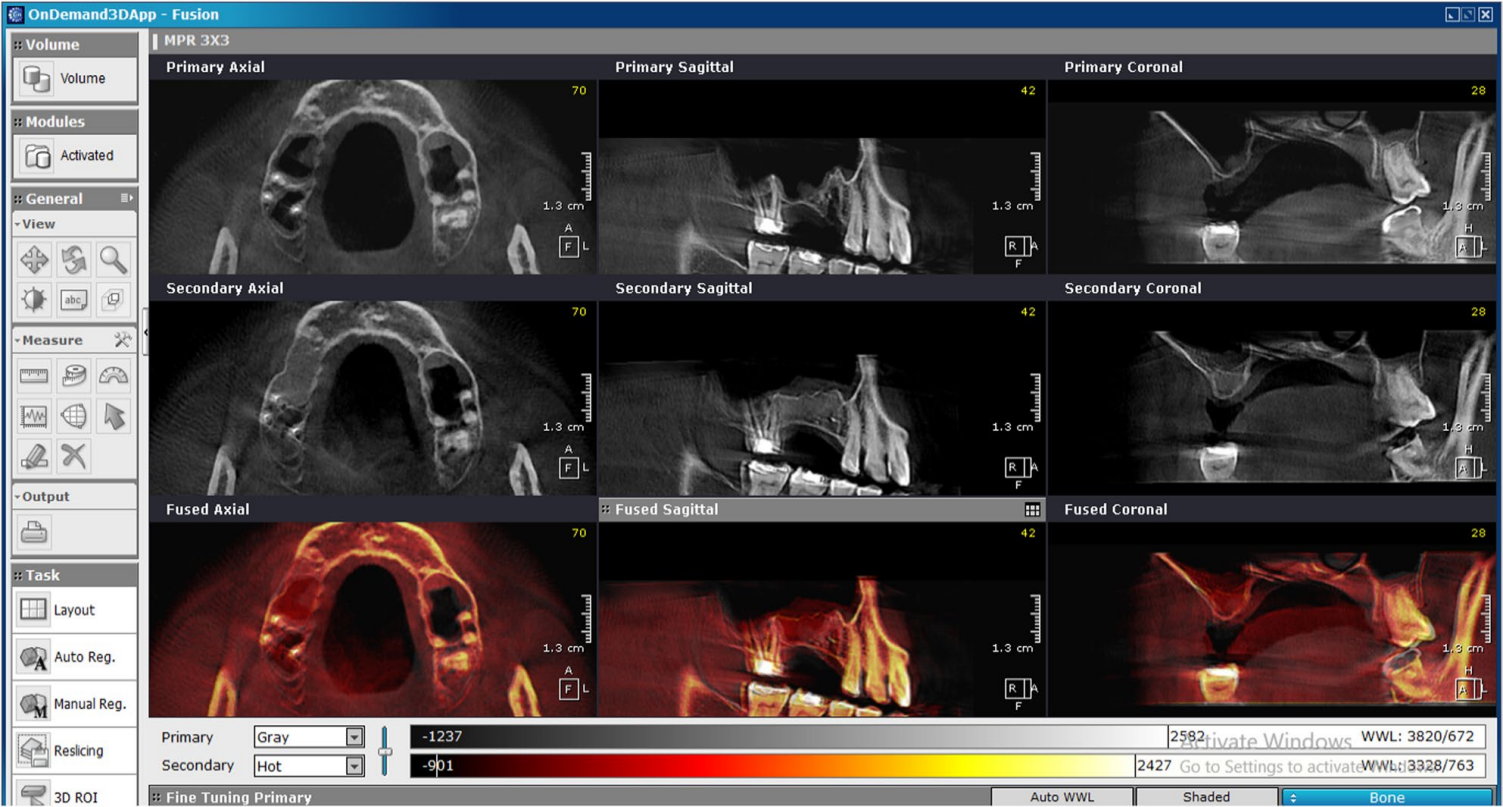
A CBCT fusion between the preoperative and 8 months postoperative showing the bone formation at the site of operation. (intervention group)

Eight months post-surgery, CBCT scans were taken using the same device and settings as preoperatively. Using OnDemand 3D software, sinus floor measurements were recorded at the highest point according to standard sinus augmentation protocols [21]. All measurements were recorded using the software’s calibrated millimeter scale (Fig. 5). Fusion between preoperative and 8-month postoperative CBCT images was performed to evaluate the degree of bone height gain after augmentation, ensuring precise alignment of anatomical landmarks and enhancing the accuracy of linear measurements. This was generated by OnDemand3D software (Cybermed Inc., Seoul, South Korea) (Fig. 6).

### Second stage surgery

**Fig. 7:**
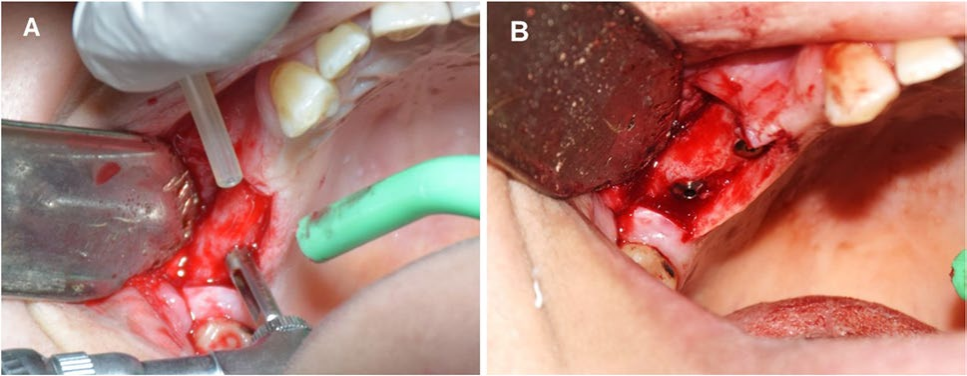
**A** Clinical photograph demonstrating the use of a trephine bur for bone core biopsy retrieval, **B** followed by final implant placement in the graftless sinus augmentation site. (intervention group)

**Fig. 8:**
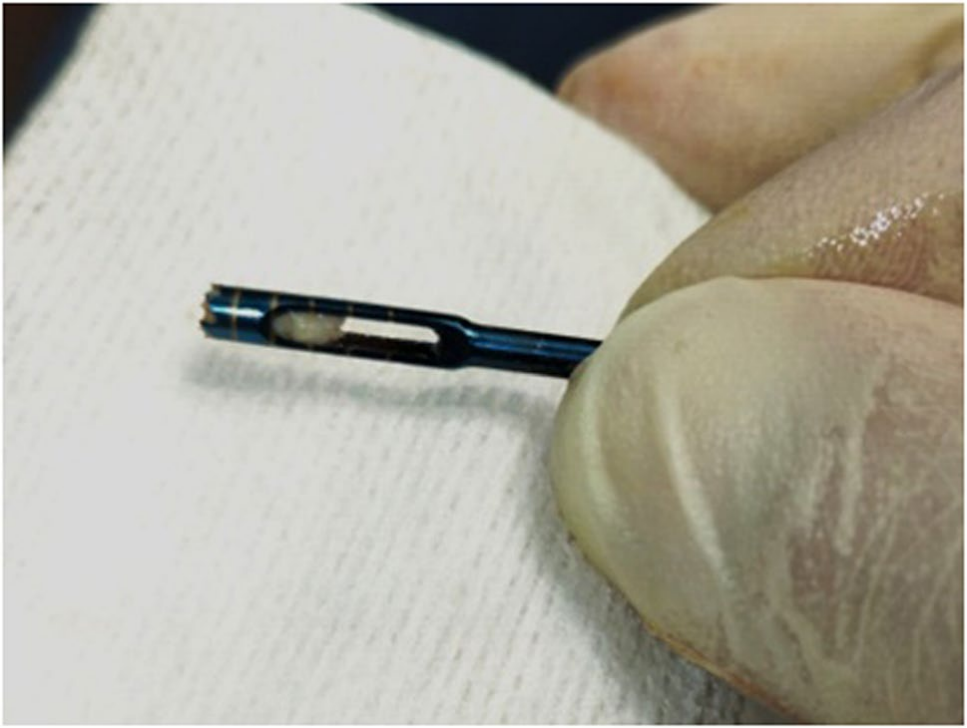
Clinical image showing a harvested bone core from the graftless sinus site using a trephine bur, prior to histomorphometric processing. (intervention group)

All surgical procedures, including core biopsy collection and subsequent implant placement, were performed under local anesthesia (Fig. 7A, B). At 8 months post-sinus elevation, core biopsies were collected using a trephine bur (outer diameter: 3.0 mm; inner diameter: 2.9 mm; length: 10 mm) (Fig. 8). The resulting osteotomy sites facilitated implant placement without the need for further drilling. The biopsy sites were guided by CBCT to ensure sampling of both native and newly formed bone. Implants were inserted with primary stability achieved in all 14 augmented sites, and no intraoperative complications were reported.

### Specimen processing

Tissue specimens were fixed in 10% buffered formalin for 72 h, decalcified in 10% diluted formic acid, and processed following a standardized laboratory protocol. They were dehydrated through ascending concentrations of ethanol, cleared in xylene, and embedded in paraffin blocks in a uniform longitudinal orientation. Serial longitudinal sections, 4 μm thick, were obtained with a rotary microtome (Leica, Wetzlar, Germany) and subsequently stained with hematoxylin and eosin (H&E) for microscopic assessment.

### Radiographic assessment

CBCT imaging (Papaya 3D Plus, Genoray Co., Korea) with an exposure factor of 90 kV, a mA of 12 and an exposure time of 14.5 was used. The orientation beam was used to adjust the jawbone parallel to the reference surface. All measurements were conducted by an external, blinded, and calibrated oral and maxillofacial radiologist utilizing OnDemand3D software. To verify measurement reliability, intra-examiner calibration was conducted by remeasuring 20% of randomly selected scans after a two-week interval, yielding an intra-class correlation coefficient (ICC) of 0.92. Consistent anatomical landmarks were used to ensure identical measurement regions of the alveolar bone crest across all scans. Linear measurements were obtained on the multiplanar reconstruction screen after aligning the reformatted panoramic and sagittal views to visualize the augmented sinus accurately. Using the software’s measurement tools, the distance from the new sinus floor to the alveolar crest was measured to determine the augmented bone height and width for implant planning.

### Histomorphometric analysis

Following fixation, the specimens were decalcified using a conventional protocol involving immersion in a decalcifying agent at room temperature with periodic solution changes and gentle agitation to expedite the process. Decalcification was performed with 10% diluted formic acid, a weak organic acid that offers an optimal balance between speed and preservation of tissue integrity. Its moderate action minimizes damage to cellular structures and nuclear staining compared with stronger acids while remaining considerably faster than chelating agents such as EDTA.

Histopathological evaluation was conducted using an HD digital camera (XCAM1080PHB) mounted on a light microscope (SOPTOP EX20, China). Decalcified sections stained with hematoxylin and eosin (H&E) were analyzed with ImageJ software (version 1.53e). For each specimen, five fields at ×10 magnification were assessed to calculate the percentage area of newly formed viable bone and bone marrow. The region of interest (ROI) was elected at the site of new bone formation, excluding irrelevant regions such as surrounding soft tissue or non-bone structures. Using the *freehand selection tool* in ImageJ, the entire bone/defect area was contoured, and the “Edit > Clear Outside” function was applied to isolate the bone region for quantitative analysis. The software then calculated the area occupied by newly formed bone as a percentage of the total ROI area. All measurements were performed by a blinded examiner, and multiple sections per sample were analyzed to ensure reproducibility and reduce measurement bias.

### Statistical methods

Statistical analysis of the results was performed using SPSS software. Shapiro-Wilk test of normality was used to test normality hypothesis of all continuous variables. Paired T-test was used for evaluation of statistical significance of bone height before and after each treatment. Unpaired T-test was used to evaluate the statistical significance of each parameter between the control group and the intervention group. P-values ≤ 0.05 were considered statistically significant.

## Results

A total of 14 patients were included in this study, 7 patients in each group. 12 females and 2 males. The mean age of all participants was 42.4 ± 7.4 years (range: 35–60 years).

### Clinical findings

No major complications were encountered during the healing phase, apart from transient postoperative pain and edema. Clinical evaluations revealed no evidence of inflammation, bleeding, or other adverse events at the surgical sites. Wound healing was complete within 7–10 days after suture removal. Patients were followed clinically at 1 week, 6 months, and 8 months postoperatively, up to the delivery of the final prosthetic restoration, with no reported signs of tenderness or sinusitis.

### Radiographic results

#### Crestal bone height (mm)

Postoperative regenerated crestal bone height demonstrated an increase from 2.54 ± 1.09 mm preoperatively to 12.07 ± 1.84 mm 8 months postoperatively in the control group. Likewise, the intervention group showed an increase in crestal bone height from 2.72 ± 0.53 mm to 8.43 ± 1.23 mm. The greatest mean of crestal bone height was recorded in the control group 8 months postoperatively. Paired T-test revealed that in both control and intervention groups, the postoperative crestal bone height was significantly higher than the preoperative height (Table 1). Statistical comparison using an unpaired t-test exposed a significant difference in postoperative crestal bone height, with the control group revealing higher values than the intervention group (Table 2).

**Table 1:** Crestal bone height in both groups and significance difference using paired T-test.*Significant at p<0.05

	Control group		intervention group	
	Preoperative (mm)	Postoperative (after 8 months) (mm)	Preoperative (mm)	Postoperative (after 8 months) (mm)
Mean	2.54	12.07	2.72	8.43
SD	1.10	1.80	0.50	1.30
Minimum	1	10	2	7.15
Maximum	4	15	3.60	10.80
P-value	< 0.00001*		< 0.00001*	

**Table 2:** Crestal bone height in the postoperative of both groups and significance of the difference using un-paired T-test

	Control group postoperative (after 8 months)	Intervention group postoperative (after 8 months)
Mean	12.07	8.43
SD	1.80	1.30
Minimum	10	7.15
Maximum	15	10.8
P-value	0.000533	

*Significant at *p* < 0.05

### Histomorphometric results

#### Area percentage of formed bone (%)

**Fig. 9:**
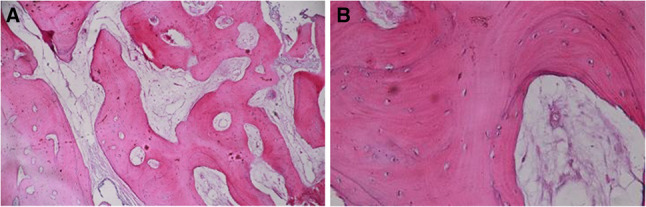
Histomorphometric images of sections taken from core biopsy of the intervention group and stained with H&E (**A**): original magnification × 100, (**B**): original magnification × 400. The bone at the intervention site is showing normal bone trabeculae and bone marrow spaces. The spaces are not showing any signs of inflammation or inflammatory infiltrate

**Fig. 10:**
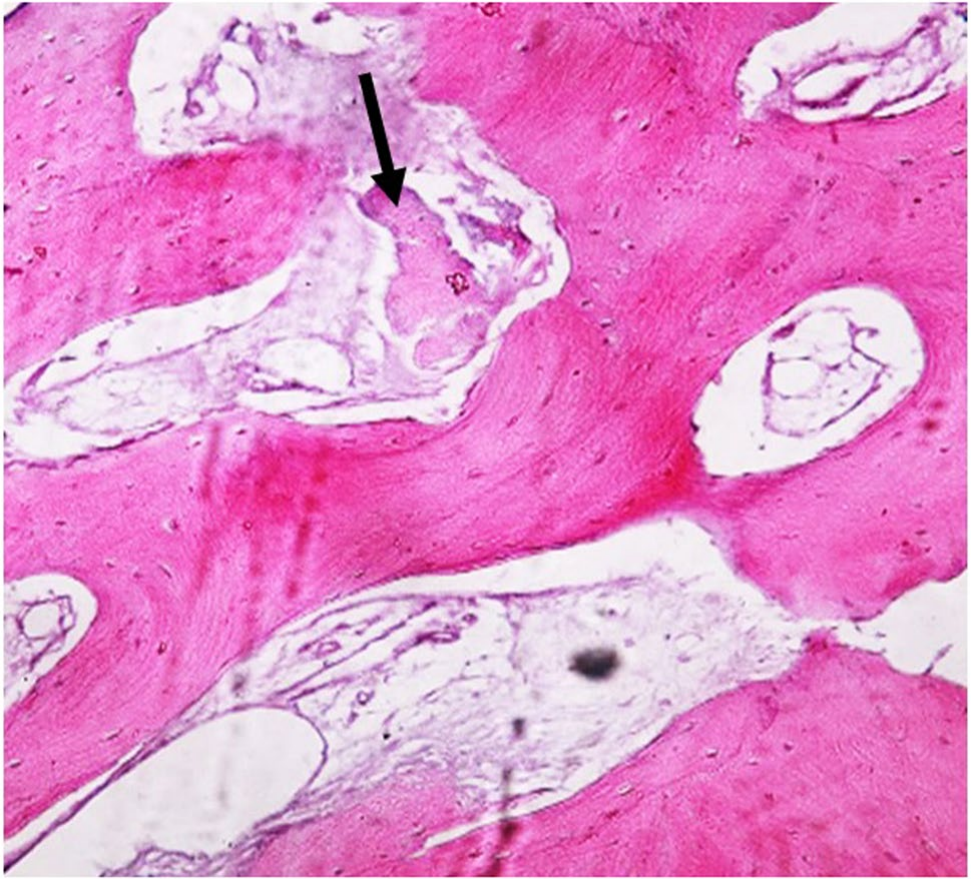
The Histomorphometric section of control group stained by H&E showing bone graft. Black arrow showed the remnants of the xenograft (Original Mag.x100)

Histomorphometric sections taken from core biopsies of all groups showed new cortical bone formation with osteoblastic rimming and bone spicules. Neovascularization in the form of small capillaries was also evident in both groups. The bone trabeculae were interconnected, and all marrow spaces were vascular as shown in (Fig. 9A& B) (Fig. 10).

Although the mean area percentage of bone formation was higher in the intervention group than in the control group, unpaired t-test results indicated no significant difference between the groups (*P* = 0.324) (Table 3).

**Table 3:** Area percent of newly formed bone in both groups and significance of the difference using unpaired T-test

	Control	Intervention
Mean	35.20%	37.30%
SD	9.01	8.90
Minimum	24%	25.10%
Maximum	42.50%	46.90%
P-value	0.324	

## Discussion

Growing evidence has revealed that the Schneiderian membrane functions as an active contributor to bone regeneration, owing to its inherent osteogenic capacity. This biological potential is evidenced by the membrane’s ability to express several osteogenic markers, including osteonectin, osteopontin, osteocalcin, alkaline phosphatase, and bone morphogenetic protein-2 (BMP-2), all of which contribute significantly to the initiation and regulation of new bone formation within the maxillary sinus [22, 23].

The present clinical study, involving 14 patients randomly allocated into two groups, aimed to evaluate new bone formation following maxillary sinus floor elevation using bioabsorbable screws as space-maintaining devices in the absence of any grafting material. The findings contribute to the growing body of evidence supporting graftless sinus augmentation techniques, which seek to simplify surgical protocols while achieving clinical outcomes comparable to those obtained with conventional grafting approaches [24, 25]. These studies have supported the feasibility of non-grafted sinus floor elevation using various space-maintaining devices.

In alignment with these findings, the current study demonstrated both radiographic and histomorphometric evidence of new bone formation following sinus membrane elevation without the use of grafting materials. Although the intervention group showed a significant intragroup increase in vertical bone height between the preoperative assessment and the 8-month postoperative evaluation, radiographic comparison revealed a statistically significant reduction in bone height when compared to the control group. However, from a histomorphometric perspective, the intervention group demonstrated a greater proportion of new bone formation relative to the control group with non-significant effect.

Bone grafts and bone substitute materials are commonly employed to facilitate successful maxillary sinus floor augmentation [26]. However, their use may be associated with several limitations, including donor site morbidity in the case of autografts and unpredictable resorption rates in allografts or xenografts both of which can negatively affect implant osseointegration. The present study supports the efficacy of a graftless sinus elevation technique utilizing bioabsorbable screws for space maintenance. This approach may eliminate the need for conventional grafting materials and their associated complications.

The results of the present study align with previous reports indicating that sinus membrane elevation alone, when adequately stabilized, can stimulate spontaneous bone formation originating from the Schneiderian membrane and the surrounding bony walls of the maxillary sinus. The inherent osteogenic potential of the sinus floor, coupled with the vascularity of the Schneiderian membrane, appears to play a critical role in bone regeneration. When a secluded and stable healing environment is achieved, it facilitates the recruitment of osteoprogenitor cells and supports the formation of new bone tissue, even in the absence of additional grafting materials [27–29].

Moreover, another study consistent with our results demonstrated successful bone regeneration in graftless sinus augmentation using a resorbable mesh as a space-maintaining device. The histomorphometric analysis in his study revealed the regenerative capacity of the Schneiderian membrane when adequately stabilized [30]. However, this study was a case series without a control group, limiting its ability to provide comparative data. In contrast, the present randomized controlled trial enabled a direct comparison between grafted and non-grafted approaches, thereby yielding more robust and generalizable evidence regarding the efficacy of space-maintaining resorbable devices in maxillary sinus augmentation [30]. Further evidence was provided by Lundgren et al., who demonstrated that bone formation could be achieved by maintaining a secluded compartment beneath the elevated membrane, using suturing and a replaceable bone window, with no additional biomaterials [28].

Among studies supporting graftless sinus augmentation, the clinical trial by Zahedpasha et al. most closely resembles the present investigation [31]. In their randomized split-mouth study, sinus membrane elevation was compared with and without bovine bone substitute (Cerabone), with simultaneous implant placement in both groups. In the graftless group, membrane stability was achieved through the tenting effect of the implants alone. Although the Cerabone group showed slightly greater radiographic bone gain, histomorphometric analysis revealed superior bone formation in the intervention group, including increased trabecular thickness and more mature, vascularized bone [31]. In contrast to their approach, our study utilized bioabsorbable screws as a mechanical means to support the elevated membrane instead of implant placement, as the alveolar bone height wasn’t enough to insert implant in the same surgery.

Magro-Filho & Gomes revealed that blood clot shrinkage of up to 43% can occur within 6 months when not adequately supported, highlighting the risk of membrane collapse and compromised bone volume [32]. Moreover, in case of completely edentulous maxillae with massive loss of alveolar bone height and huge space, it may lead to a less stable blood clot and futured callus formation particularly if there is no bone graft which can act as a scaffold in this created space [33]. Lie et al. reported that while implant survival rates were comparable between grafted and graftless procedures (98%), the latter exhibited significantly lower vertical bone gain (1.7 mm less) and reduced bone density (95 HU) [34]. A systematic review and meta-analysis of randomized controlled trials reported that graftless sinus lift procedures resulted in significantly less vertical bone height gain compared to grafted techniques. However, implant survival rates remained high and comparable between the two groups (97.9% vs. 98.7%) [35].

Another study done by Scala et al. clarified the importance of tenting where unsupported Schneiderian membrane can lead to collapses and limiting the amount of bone gain, which mandates the importance of space maintainers to allow bone formation [36]. This result explained our results of less bone height gained in the intervention group than the control group. In the present study, the use of biodegradable screws resulted in lower vertical bone height after 8 months compared to the xenograft-augmented group. This result was supported by the concept of collapse that occurred by the action of pneumatization and the negative pressure inside the sinus. Therefore, stabilizing the blood clot has become a necessity to ensure predictable bone formation. In this context, our study proposes the use of bioabsorbable screws as a novel approach to provide stable membrane elevation while eliminating the need for a second surgery for hardware removal.

The main advantage of the Bioabsorbable screws is the biodegradability, thus hindering further surgery to remove them. Unlike titanium or other non-resorbable devices, Bioabsorbable screws eliminate the need for a secondary procedure for removal, reduce the risk of chronic inflammation, and eventually integrate with the surrounding tissues.

Animal and clinical investigations have reported that screws fixed in bone exhibit a strength level similar to titanium screws [30]. Bioabsorbable screws have been used in graft fixation, osteosynthesis, and distraction [37]. Moreover, the use of Bioabsorbable screws offers another clinical advantage which is the space-maintaining function, while temporary, is sufficient to support the critical early phase of healing and bone formation.

The success of space maintenance relies on the timing of screws degradation. Premature disintegration may hinder osteogenesis; therefore, the resorption period should extend beyond the time required for new bone formation. However, the exact duration necessary for bone regeneration or pneumatization beneath an unsupported elevated Schneiderian membrane remains uncertain.

In the current study, histomorphometric analysis 8 months post-surgery revealed well-vascularized, newly formed bone in both groups, with characteristic osteoblastic rimming, interconnected bone trabeculae, and absence of inflammatory infiltration. The area percent of new bone was higher in the intervention group (mean: 37.3%) compared to the control group (35.2%), though this difference was not statistically significant (*P* = 0.324).

These findings align closely with the results of Zahedpasha et al., who conducted a split-mouth study comparing sinus membrane elevation with and without grafting. Their histomorphometric evaluation showed mature, Haversian-like bone structures in the graftless sites, with more organized trabeculae and less inflammation than the grafted group [31].

Despite the widespread use of xenograft materials such as deproteinized bovine bone in sinus augmentation procedures, their long-term persistence within the grafted site can hinder optimal bone regeneration. The current study demonstrated a higher with no significance percentage of newly formed bone in the intervention group compared to the control group. The retention of foreign graft particles may negatively influence bone healing by impairing normal remodeling mechanisms, resulting in compromised bone quality and ultimately reducing the effectiveness of osteogenesis. Supporting our findings, Kudsi et al. conducted a comparative clinical and histomorphometric study examining the outcomes of bovine bone xenograft (Bio-Oss). Their results demonstrated that 40% of the xenograft group showed no evidence of mineralized tissue formation five months after surgery. Moreover, all samples from the xenograft group displayed residual graft particles, and a significant reduction in bone height was observed compared to their control group [38]. These findings align with our observations and highlight a key limitation of xenograft materials, namely, their slow resorption rate and tendency to remain in situ without contributing meaningfully to new bone formation.

The results of the present study are supported by a systematic review and meta-analysis by Chen et al., which assessed maxillary sinus floor elevation using grafted and non-grafted techniques via both internal and external approaches. Their evaluation of 17 randomized controlled trials revealed no significant difference in short-term implant survival between graftless and conventionally grafted sinus augmentation procedures [39].

Moreover, the findings of the present study are in agreement with the randomized clinical trial conducted by Aboul Fettouh et al., who evaluated graftless maxillary sinus floor elevation using either the lateral window or transcrestal approach with a one-year follow-up. Their results demonstrated that both techniques achieved comparable implant stability and marginal bone level outcomes, despite the absence of grafting materials, supporting the concept that the Schneiderian membrane and blood clot alone can promote new bone formation [40].

The biological advantage of this graftless approach was reflected in the histomorphometric quality of the regenerated bone and its potential to support subsequent implant integration. Further studies with larger cohorts and long-term evaluation are recommended to validate clinical success and implant outcomes.

A key limitation of the present study is that, although membrane elevation was successfully achieved, the use of bioabsorbable screws did not provide space maintenance comparable to the xenograft control, as demonstrated by the significantly lower vertical bone height gain observed in the intervention group. This reduced gain may be attributed to membrane collapse and variability in screw resorption behavior. Nevertheless, histological analysis confirmed successful new bone formation in the intervention group, indicating that osteogenesis occurred despite the reduced vertical height gain. These findings suggest that while the graftless approach supported bone regeneration at the histological level, mechanical stability and space maintenance remain critical factors influencing radiographic outcomes. Additional limitations include the relatively small sample size and limited follow-up period. Future studies with larger cohorts, longer-term evaluation, and alternative or adjunctive stabilization methods are needed to optimize both the quantity and stability of regenerated bone augmentation.

## Conclusion

This study suggests that successful bone regeneration in graftless sinus augmentation is achievable when the Schneiderian membrane is properly stabilized. Bioabsorbable screws provided effective support, aiding clot preservation and bone organization without triggering inflammation as shown by histomorphometrical findings. This approach offers a low-morbidity alternative to grafting and eliminates the need for hardware removal.

## Supplementary Information

Supplementary Material 1

Supplementary Material 2

Supplementary Material 3

## Data Availability

The de-identified participant data and statistical code used in this study are available from the corresponding author upon reasonable request.

## Abbreviations

RCTs: Randomized Controlled Trials
CBCT: Cone Beam Computed Tomography
DBBM: Deproteinized Bovine Bone Mineral
H&E: Hematoxylin and Eosin
mm: Millimetre
ICC: Intra-class Correlation Coefficient
