# Supplementary material for: Clinical, radiographic and histomorphometry evaluation of the osteogenic potential of Schneiderian membrane after graftless maxillary sinus augmentation: a randomized controlled clinical trial
Source: BMC Oral Health. 2026 Feb 19;26:417. doi: 10.1186/s12903-026-07772-2 (PMC12954954; doi:10.1186/s12903-026-07772-2)
Supplement: Supplementary file 1 — Supplementary Material 1 [file 12903_2026_7772_MOESM1_ESM.pdf]

ClinicalTrials.gov Protocol Registration and Results System (PRS) Receipt
Release Date: August 7, 2025

ClinicalTrials.gov ID: NCT06766292

Study Identification

Unique Protocol ID: 672/2023

Brief Title: Osteogenic Potential of Schneiderian Membrane

Official Title: Clinical, Radiographic and Histologic Assessment of the Osteogenic Potential of Schneiderian Membrane After Maxillary Sinus Lifting: A Randomized Controlled Clinical Trial.

Secondary IDs:

Study Status

Record Verification: August 2025

Overall Status: Completed

Study Start: August 27, 2023 [Actual]

Primary Completion: August 3, 2024 [Actual]

Study Completion: September 3, 2024 [Actual]

Sponsor/Collaborators

Sponsor: rehab soliman

Responsible Party: Sponsor-Investigator
Investigator: rehab soliman [rehab soliman]
Official Title: lecturer of oral and maxillofacial surgery
Affiliation: Ain Shams University

Collaborators:

Oversight

U.S. FDA-regulated Drug: No

U.S. FDA-regulated Device: No

U.S. FDA IND/IDE: No

Human Subjects Review: Board Status: Approved
Approval Number: 672/2023
Board Name: research ethics committee
Board Affiliation: suez canal university faculty of dentistry
Phone: 0643230210
Email: president_office@suez.canal.eg
Address:

4.5km ring road, Ismailia, Egypt

Data Monitoring: No

Study Description

Brief Summary: Maxillary sinus augmentation is commonly performed by creating a space below the Schneiderian membrane which is further filled using autografts, bone graft substitutes, or a combination of both to allow for new bone formation. Recently, a new method called non-grafting sinus floor augmentation has been introduced as an alternative approach. This method is based on the idea of elevating the membrane and supporting it through implant insertion or the use of space-maintaining devices like titanium screws or mesh. This study was conducted to evaluate the efficacy of new bone formation after sinus floor elevation using space maintaining resorbable pins without graft material.

Detailed Description: A randomized control trial was performed on 14 patients with partially edentulous or free end saddle posterior maxilla. The patients were divided randomly into two groups. Both groups underwent maxillary sinus open lifting procedure and augmentation using xenograft * as group A and in group B the sinus membrane was elevated and stabilized by resorbable pins** to maintain the created space stable and without grafting. The study was a double blind one (participants and out¬come assessors were blinded throughout the study).

All the surgical procedures were carried out un¬der local anesthesia using infraorbital and posterior superior alveolar nerve block with palatal infiltration. Full thickness mucoperiosteal flap was elevated to expose the lateral wall of the maxillary sinus. Then the maxil¬lary sinus floor elevation using the lateral window technique was performed. A bone window was outlined using a no. 8 diamond bur mounted on straight hand piece with copious irrigation (sterile saline solution) with cautious taken to not penetrate the sinus membrane. The process of bone removal was done through the cortical bone to reach the membrane without perforation. Complete osteotomy along the boundary of the osseous window until the Schneiderian mem-brane. The Schneiderian membrane was carefully elevated till the desired height.

xenograft Preparation: (control group/ group A)

Xenograft was mixed with saline followed by the protocol of graft packed and compacted against inferior walls and to the sides of the antrum until the new available volume created was filled. The lateral window was covered by collagen membrane before flap closure. Resorbable pins (test group/ group B):

In the study group: 2.0 mm screws were fixed to the lateral wall of the sinus above the superior osteotomy and placed buccopalatally where the schneiderian membrane was elevated and maintained. Then the soft tissue flap was readapted and sutured using continuous and interrupted sutures (3-0 resorbable vicryl)

Conditions

Conditions: Bone Healing
Sinus Membrane Osteoginicity

Keywords: sinus lifting
maxillary sinus augmentation

Study Design

Study Type: Interventional

Primary Purpose: Treatment

Study Phase: N/A

Interventional Study Model: Parallel Assignment

Number of Arms: 2

Masking: Triple (Participant, Investigator, Outcomes Assessor)

Allocation: Randomized

Enrollment: 14 [Actual]

Arms and Interventions

Arms	Assigned Interventions
Active Comparator: Control Group<br>in this group, the patients underwent open sinus lifting surgery and augmentation of the sinuses by xenograft without any additives	Procedure/Surgery: open sinus lifting with xenograft only<br>ll Patients in this group underwent open sinus procedures to lift the sinus membrane followed by application of the xenograft as an augmentation material xenograft is used instead of autogenous bone graft to avoid the morbidity of the donor side
Experimental: study group<br>In the study group: 2.0 mm screws were fixed to the lateral wall of the sinus above the superior osteotomy and placed buccopalatally where the schneiderian membrane was elevated and maintained.	Procedure/Surgery: primary intervention<br>In the study group: 2.0 mm screws were fixed to the lateral wall of the sinus above the superior osteotomy and placed buccopalatally where the schneiderian membrane was elevated and maintained. this technique will suspends the membrane in position to allow the blood clot to organized and formation of bone.

Outcome Measures

Primary Outcome Measure:

1. bone height
   will be measured using CBCT (Cone Beam Computed Tomography)

   [Time Frame: 8 months]

Secondary Outcome Measure:

2. histological analysis
   will be measured using hematoxylin and eosin stains

   [Time Frame: 8 months]

Eligibility

Minimum Age: 25 Years

Maximum Age: 55 Years

Sex: All

Gender Based:

Accepts Healthy Volunteers: Yes

Criteria: Inclusion Criteria:

1. Adult male/female patients above the age of 25.
2. Patients with one or more teeth requiring implant supported dental restoration in atrophic maxilla (unilateral or bilateral).
3. Alveolar bone height less than 4 mm at the defective site.
4. Good oral hygiene.

Exclusion Criteria:

1. Medically compromised patients with conditions contraindicating surgery (eg. uncontrolled diabetics, bisphosphonate intake, radio or chemotherapy).
2. Patients with active infection at or related to the site of surgery (eg. acute sinusitis).
3. Heavy smokers.
4. Patients not indicated for an implant supported restoration at the time of enrollment (eg. active/untreated periodontal disease).

Contacts/Locations

Central Contact Person: rehab A soliman, lecturer
Telephone: +201018662326
Email: rehabsoliman4@gmail.com

Central Contact Backup: Inas A Helwa, lecturer
Telephone: +201033333600
Email: Inas.helwa@miu.egypt.edu.eg

Study Officials: rehab soliman
Study Principal Investigator
Misr International University

Locations: **Egypt**
suez canal university
Ismailia, Egypt, 41522
Contact: rehab A soliman, lecturer +201018662326
rehabsoliman4@gmail.com
Contact: randa A Mokhtar, lecturer +201006382072
randa.amin@miuegypt.edu.eg
Principal Investigator: Mohamed A Elsholkamy, professor
Sub-Investigator: randa A Mokhtar, lecturer
Sub-Investigator: Inas A Helwa, lecturer

IPDSharing

Plan to Share IPD: No

References

Citations:

Links:

Available IPD/Information:
