# Supplementary material for: Clinical, radiographic and histomorphometry evaluation of the osteogenic potential of Schneiderian membrane after graftless maxillary sinus augmentation: a randomized controlled clinical trial
Source: BMC Oral Health. 2026 Feb 19;26:417. doi: 10.1186/s12903-026-07772-2 (PMC12954954; doi:10.1186/s12903-026-07772-2)
Supplement: Supplementary file 2 — Supplementary Material 2 [file 12903_2026_7772_MOESM2_ESM.docx]

# Original Article: Clinical Trial

Clinical, Radiographic and Histomorphometry evaluation of the Osteogenic Potential of Schneiderian Membrane after graftless Maxillary Sinus augmentation: A Randomized Controlled Clinical Trial

Authors:

Mohamed ElSholkamy^1^, Randa H. Mokhtar^2^, Tarek M. Eltayeb^3^, Inas Helwa^2^, Rehab A. Soliman^4^*

Affiliations:

^1^ Oral & Maxillofacial Surgery Department, Faculty of Dentistry, Suez Canal University, Ismailia, Egypt

^2^ Oral Histopathology Department, Faculty of Oral and Dental Medicine, Misr International University, Cairo, Egypt

^3^ Oral Medicine, Periodontology and Oral Diagnosis Department, Faculty of Oral and Dental Medicine, Misr International University, Cairo, Egypt

^4^ Oral & Maxillofacial Surgery Department, Faculty of Oral and Dental Medicine, Misr International University, Cairo, Egypt

ORCID IDs:

Mohamed ElSholkamy: 0009-0000-0367-6573

Randa H. Mokhtar: 0000-0001-9185-0403

Inas Helwa: 0000-0002-9294-9277

Tarek M. Eltayeb: 0000-0001-5500-1658

Rehab A. Soliman: 0000-0002-9713-8510

Corresponding Author:

Rehab A. Soliman

Email: rehab.abdallah@miuegypt.edu.eg

Lecturer, Oral & Maxillofacial Surgery Department, Faculty of Oral and Dental Medicine, Misr International University, Cairo, Egypt.

Address: 19 taj-city, Cairo, Egypt
